# Pigtail saves a twisted redo: successful management of chylopericardium after midline valve surgery

**DOI:** 10.15171/jcvtr.2018.09

**Published:** 2018-03-17

**Authors:** Aayush Goyal, Rohit Shahapurkar, Balaji Aironi, Ninad Kotkar, Ankur Goel

**Affiliations:** P K Sen Department of Cardiovascular and thoracic Surgery, Seth GS Medical College and KEM Hospital, Parel, Mumbai, India.

**Keywords:** Chylopericardium, Chylomediastinum,, Complication Valve Replacement

## Abstract

Pericardial effusion after midline cardiac surgery may be transudative or exudative. The exudative infective or haemorrhagic variety requires early surgical intervention. However there are rare cases of collections like chylomediastinum which should be ruled out. Their low incidence prompts to establish protocol for evaluating postoperative pericardial collections, which includes echocardiography and biochemical analysis of aspirate. The same is important from the perspective of management as chylopericardium may be successfully managed without surgical intervention by aspiration, pig tail insertion, dietary and medical management, which we demonstrate through our rare case which occurred after midline double valve replacement.

## Introduction

Symptomatic pericardial effusion after month of midline cardiac surgery is an emergency, mostly fearing infective or hemorrhagic collection, more so in undereducated patients on anticoagulation with underprivileged unhygienic living conditions, not so rare in developing countries. Although transudative pericardial effusion either reactionary or because of cardiac failure should always be investigated, there are other causes, which must be ruled out before embarking the invasive way back into the mediastinum. Chylo-pericardium is a rare cause of pericardial collection after a midline valve surgery, even in absence of any excessive dissection in mediastinum, neck or around left superior vena cava or breach in pleura. There are few case reports highlighting their anecdotal incidence and their management strategy.1 We report chylo-mediastinum in a young patient 2 months after double valve replacement, and highlight the importance of systematic investigation and successful non-surgical management.

## Case Report

**Figure 1:**
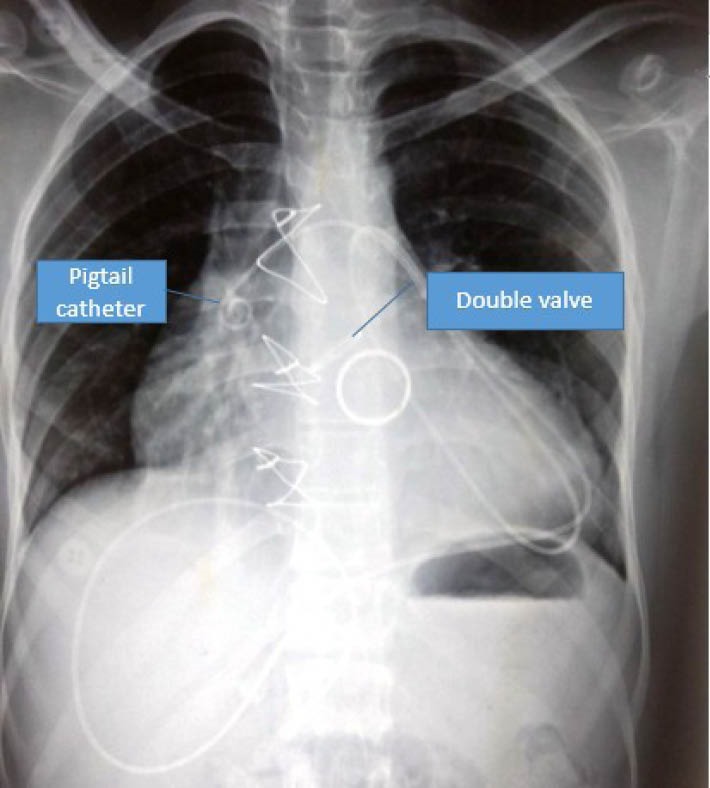
X-Ray demonstrating Pig Tail catheter in the Pericardial Cavity. Both Aortic and Mitral valve are visible. No evidence of pleural collection.

A 24-year-old shopkeeper from rural India, was symptomatic for severe rheumatic mitral stenosis since the age of 16 years and underwent balloon mitral valvotomy in 2009. He was on medical management and regular follow up when he became symptomatic in January 2017 with NYHA class II Dyspnoe on exertion progressing to class III. He was euthyroid and had no other co morbid medical or surgical illness. Echocardiography was suggestive of severe aortic stenosis and severe mitral stenosis and underwent double valve replacement with Sorin Bicarbon 27 mm bileaflet mitral prosthetic valve and St Jude Medical Regent 21 mm bileaflet aortic prosthetic valve in April 2017 through midline sternotomy. Pleura were not breached during the procedure. Patient required mediastinal lavage on post-operative day three in view of mediastinitis. He further required bilateral pleural aspiration on postoperative day five for pleural collection, which was serous in nature with no growth on culture. The patient recovered uneventfully and discharged with echochardiography suggestive of adequate valve function, no pericardial collection and no evidence of pleural collection. The patient became symptomatic again after two months with progressive dyspnoe on exertion class III. There was no history of fever but a documented weight loss of 5 kg. On investigations he was diagnosed to have large pericardial collection with preserved prosthetic valve and myocardial function, Right Atrium and Right Ventricle showed diastolic collapse. There was no pleural collection. On pericardial aspiration, 900 ml of chylous fluid was drained and pigtail catheter was secured in pericardial cavity (Figure 1). Fluid analysis revealed ADA of 8.6 IU/L (Normal 0-30 IU/L), Triglyceride 1500 mg/dL (Normal <20 mg/dL), Protein 5.2 with WBC count of 2900 per cubic cm, 95% being lymphocytes. The patient was managed on medium chain triglyceride (MCT) diet and by day two, the drainage reduced to zero. The culture of the aspirate did not grow any organism. The pigtail removed and the patient was discharged after ten days with echocardiography suggestive of no pericardial collection. Follow up after one month revealed no pericardial collection with a weight gain of 3 kg.

## Discussion

Chylopericardium or the collection of chyle in the pericardium, post valve replacement through midline sternotomy is sporadically reported.1-3 Chylous fluid leak significant to cause collection is indicative of thoracic duct injury or one of its major channels. Thoracic duct, classically is not encountered in operative field during valve replacement. Mechanisms causing this rare complication are not confirmed and proposed to be due to traction on the duct from manipulation of the heart and great vessels. Rarely thrombosis at the junction of the left jugular and subclavian veins may obstructs thoracic duct drainage.1 Very rarely connections develop between the pericardial sac and a lymphatic leak.1 Delayed presentation is suggestive of slow accumulation of chyle supporting the hypothesis of opening up of new lymphatic connections.4 The diagnosis of chylopericardium is confirmed by chemical analysis of drained chylous milky fluid cholesterol level greater than 110 mg/dL, a cholesterol-to-triglycerides ratio of less than 1 and a triglyceride level greater than 500 mg/dL. It is hard to extrapolate that intervention in form of mediastinal lavage or pleural aspiration could have resulted in the chylopericardium in our patient. Can repeated sternal wire closure in a setting of mediastinitis inadvertently causes lymphatic injury to cause the collection is a farfetched assumption. Although it should be investigated whether infection or mediastinitis is an etiology as many of the cases reported have had either intervention for infection, carditis or developed early post operative infection or reintervention1,5 which incidentally was in our case as well.

The management can be medical or surgical. The site of culprit leak can be investigated with the help of computer tomography based lymphangiography, invasive angiography or nuclear lymphoscintigraphy. This also quantifies the leak and guides in appropriate management once the leak does not decrease with conservative medical management. We did not consider the diagnostic imaging as our patient responded to medical management within 48 hours. Medical management includes fluid and electrolyte management, parentral nutrition or Medium Chain triglyceride diet, somatostatin infusions.6 Surgically creation of a pericardial window tides over the crisis but definite surgical management is the ligation of thoracic duct above the diaphragm preventing repeated leaks, while the chyle gets diverted through other channels. A combination of medical management and pericardial drainage either multiple aspirations or pigtail may be done if there are facilities of repeated evaluation and round the clock operation facilities for surgical intervention if required. This ‘watchful masterly inactivity’ proved successful in our case and avoided an invasive surgical intervention.

A pericardial collection after a midline surgery large enough to cause symptoms is generally a warning sign. It must be investigated with a protocol of radiological investigation and cytological analysis after stabilizing the patient clinically. Transudative pericardial effusion either reactionary or because of cardiac failure should always be ruled out and medical management initiated, an infective or hemorrhagic collection might require an urgent surgical intervention, rare collections of chyle should be kept in mind which can be successfully managed without reentering the mediastinum surgically, not only putting the prognosis of patient in better perspective but also conserving the much needed resources in a developing country

## Ethical approval

An informed consent was obtained for publication this case.

## Competing interests

All authors declare no competing financial interests exist.
